# FGF-23, hsCRP, Cardiovascular Events, and the Benefit of Canagliflozin in the CANVAS Trial

**DOI:** 10.1016/j.jacadv.2025.102276

**Published:** 2025-10-27

**Authors:** Aranya Punithan, Ehsan Ghamarian, Daniela Grothe, Macy MacLean, Kim Connelly, Bruce Neal, Alanna Weisman, David Z.I. Cherney, Filio Billia, Jacob A. Udell

**Affiliations:** a Department of Pharmacology and Toxicology, University of Toronto, Toronto, Ontario, Canada; b Cardiovascular Division, Women’s College Hospital, Toronto, Ontario, Canada; c Applied Health Research Centre, Toronto, Ontario, Canada; d Peter Munk Cardiac Centre, University Health Network, Toronto, Ontario, Canada; e Keenan Research Center for Biomedical Science, Li Ka Shing Knowledge Institute and the Division of Cardiology, St. Michael’s Hospital, Toronto, Ontario, Canada; f Department of Medicine, University of Toronto, Toronto, Ontario, Canada; g UNSW Sydney, Sydney, New South Wales, Australia; h Lunenfeld-Tanenbaum Research Institute, Mount Sinai Hospital, Toronto, Ontario, Canada; i ICES, Toronto, Ontario, Canada; j Division of Nephrology, University of Toronto, Toronto, Ontario, Canada; k Toronto General Hospital Research Institute, University Health Network, Toronto, Ontario, Canada; l Ted Rogers Centre for Heart Research, Toronto, Ontario, Canada

**Keywords:** canagliflozin, cardiovascular outcomes, FGF-23, hsCRP, SGLT2 inhibitor

## Abstract

**Background:**

The CANVAS (Canagliflozin Cardiovascular Assessment Study) trial provided the opportunity to determine the utility of measuring cardiorenal biomarkers, such as fibroblast growth factor 23 (FGF-23) and high-sensitivity C-reactive protein (hsCRP) levels for determining risk prediction and treatment response to sodium glucose co-transporter 2 inhibitor therapy in patients with type 2 diabetes mellitus.

**Objectives:**

The prognostic value of these biomarkers for predicting adverse cardiovascular (CV) outcomes and treatment response was assessed.

**Methods:**

Of 4,330, 3,188 (73.6%) participants had available longitudinal biomarker samples. The association between FGF-23 and hsCRP with composite CV death or hospitalization for heart failure (HHF), HHF, CV death, and major adverse CV events were assessed using multivariable Cox proportional hazard models adjusted for clinical risk factors, and markers of cardiac and renal injury. Event rates by randomized treatment assignment were calculated for FGF-23 and hsCRP after assignment to a “low-risk” (quartiles [Q] 1-3) or “high-risk” (Q4) group. Multimarker risk assessment was done by stratifying participants by both FGF-23 and hsCRP quartiles to create 4 risk groups.

**Results:**

When compared with Q1, FGF-23 levels in Q4 were significantly associated with CV death/HHF (HR: 1.65; 95% CI: 1.15-2.40; *P* = 0.008) and HHF (HR: 1.96; 95% CI: 1.04-3.69; *P* = 0.037) whereas hsCRP levels in Q4 were significantly associated with CV death (HR: 1.78; 95% CI: 1.16-2.73; *P =* 0.008) and major adverse CV events (HR: 1.35; 95% CI: 1.02-1.78; *P =* 0.038) in adjusted analyses. There was consistent effect of canagliflozin vs placebo across high- and low-risk groups (*P-*interactions ≥0.30).

**Conclusions:**

FGF-23 and hsCRP are biomarkers associated with increased CV risk, but these markers did not identify participants who preferentially benefited from treatment with canagliflozin.

In general, the uptake of sodium glucose co-transporter 2 inhibitor (SGLT2i) remains slow. It has been postulated that there is a more efficient method for identifying those at the highest risk for adverse outcomes that may particularly benefit from this treatment strategy among the broad population of patients with type 2 diabetes mellitus (T2DM) and cardiovascular disease (CVD) or multiple risk factors. Such an approach may provide more personalized risk stratification and treatment decision and ultimately be more cost-effective. One method for identifying patients at higher subclinical risk is by measuring biomarkers of inflammation and mineral metabolism dysregulation which may be present years upstream of overt clinical manifestations of disease.^1^, ^2^, ^3^ Such biomarkers include fibroblast growth factor (FGF)-23, a biomarker that captures early distortion of mineral metabolism homeostasis, and high-sensitivity C-reactive protein (hsCRP), a biomarker of inflammation.^1^^,^^4^^,^^5^ As there are only a few clinical risk factors at baseline that reliably predict clinical response in patients with T2DM initiated on SGLT2i therapy, an integrated analysis incorporating these cardiorenal biomarkers may provide additional insight into the pathophysiologic process and the mechanism of action of SGLT2i. Moreover, identify patients most likely to benefit from SGLT2i therapy.

CANVAS (Canagliflozin Cardiovascular Assessment Study) randomized 4,330 participants with T2DM that have a history of or were at a high risk of atherosclerotic CV disease (ASCVD) with canagliflozin, an SGLT2i or matching placebo and followed these participants for a mean of 307 (±57) weeks.^1^ Participants treated with canagliflozin experienced a reduced risk for the composite primary outcome of death by CV causes, nonfatal myocardial infarction (MI), or nonfatal stroke.^1^ Canagliflozin was also shown to reduce the risk of hospitalization for heart failure (HHF), albuminuria progression, and decline in kidney function.^6^ The CANVAS trial therefore provided a unique opportunity to investigate the utility of measuring cardiorenal biomarkers for determining risk prediction and treatment response to SGTL2i in patients with T2DM.

## Materials and methods

### Study population

For this post hoc analysis, stored plasma samples obtained during the CANVAS trial were used. The design, findings, and outcomes of the CANVAS trial have been previously published.^6^ The CANVAS trial was a randomized, double-blind, placebo-controlled, multicenter study that evaluated the effects of canagliflozin compared with placebo on CV and renal outcomes in 4,330 enrolled participants with T2DM and a history of, or high risk for, ASCVD. Enrolled participants were randomly assigned to receive either a 100 mg dose of canagliflozin, a 300 mg dose of canagliflozin, or a matching placebo by a 1:1:1 ratio. Both doses of canagliflozin were pooled into one arm due to the similar treatment effects for these analyses. All participants provided written informed consent, and approval for the study protocol was given by all relevant Institutional Review Boards as well as the participating site’s ethics committees. The present biomarker secondary analysis was approved by the Women's College Hospital and University Health Network Institutional Review Boards.

### Outcomes of interest

The primary outcome of interest for this analysis was the composite of time to first nonfatal HHF or CV death. The secondary outcomes of interests were the individual components of the primary outcome, specifically HHF (including patients with HF at baseline) and CV death alone, as well as a composite atherosclerotic-focused outcome of major adverse cardiovascular events (MACE). MACE was defined as the composite of CV death, nonfatal stroke, and nonfatal MI.

### Biomarker assessment

Stored blood plasma samples were collected at baseline, 52, 156, and 312 weeks during the CANVAS trial to analyze biomarkers at various time points. Samples were shipped to the Peter Munk Cardiac Centre for analyses, where thawed frozen samples were tested for the markers of interest. FGF-23 was measured using a second-generation Human FGF-23 (C-Term) enzyme-linked immunosorbent assay (ELISA) kit (Immutopics, Inc). The coefficients of variations observed for intra-assay precision were 2.4% at 33.7 reference units (RU)/mL and 1.4% at 402 RU/mL. The coefficients of variations observed for inter-assay precision were 4.7% at 33.6 RU/mL and 2.4% at 293 RU/mL. hsCRP levels were measured using the DBC hsCRP ELISA kit which used a 2-step capture or sandwich-type assay (DBC-Diagnostic Biochem Canada Inc). The observed intra-assay coefficients of variations were 15.2% at 205.8 ng/mL, 5.0% at 769.2 ng/mL, and 8.3% at 8,437.8 ng/mL. The observed inter-assay coefficients of variations were 9.9% at 227.0 ng/mL, 9.5% at 1,022.2 ng/mL, and 7.8% at 8,791.8 ng/mL.

### Statistical analysis

All patients with available FGF-23 and hsCRP levels were included in the current analysis. The distribution of both FGF-23 and hsCRP was skewed and therefore was log-transformed for normalization. The original nonparametric levels were categorized into quartiles for the purpose of reporting and interpreting. Baseline patient characteristics were summarized by quartiles of FGF-23 and quartiles of hsCRP. Baseline continuous variables were described as medians and IQRs and categorical variables were described as frequencies and percentages. Comparison of baseline characteristics was made using linear regression for continuous variables, logistic regression for binary variables, and multinomial regression for categorical variables.

Adjusted estimates of the association between baseline FGF-23 and hsCRP, individually, with outcomes of interest were assessed using multivariable Cox proportional hazard models. These analyses were done with FGF-23 and hsCRP modeled as a categorical variable (quartile [Q]1: lowest concentrations; Q4: highest concentrations) with the first quartile used as reference for the HR in each quartile, and as a continuous variable (log-transformed based on right-skewed distribution). The HR per doubling (log2) of baseline FGF-23 and baseline hsCRP were also assessed using multivariable Cox proportional hazard models. Back transformation was used to estimate the HR by exponentiating the survival analysis output. To assess the effect of the step-wise addition of covariates on the association of FGF-23 or hsCRP with the outcomes of interest, 6 models were built. Model 1 adjusted for randomized treatment assignment (placebo or canagliflozin). Model 2 adjusted for covariates of model 1 in addition to age and sex. Model 3 adjusted for covariates of model 2 in addition to race, history of prior MI, stroke, HF, type 2 diabetes duration, smoking, hypertension, body mass index, high-density lipoprotein, low-density lipoprotein (LDL), and estimated glomerular filtration rate (eGFR). Model 4 adjusted for covariates of model 3 in addition to urine albumin-to-creatine ratio (UACR). Model 5 adjusted for covariates of model 4 and the reciprocal biomarker (log-transformed). Model 6 adjusted for covariates of model 5 in addition to log-transformed high-sensitivity cardiac troponin T (hs-cTnT) and log-transformed N-terminal pro–B-type natriuretic peptide (NT-proBNP). Log-rank tests were used to estimate the P trends from quartiles 1 to 4 for FGF-23 and hsCRP for the multivariable Cox proportional hazard models.

Metrics of discrimination (Harrell’s C-statistic) were calculated for the addition of FGF-23 alone, hsCRP alone, and the addition of both biomarkers to the clinical model which includes the clinical variables in the multivariate model. Spearman correlation coefficients were calculated among FGF-23, hsCRP, eGFR, UACR, hs-cTnT, and NT-proBNP to assess the direction and strength of associations between variables.

Three-year Kaplan-Meier event rates were calculated for the primary endpoint, its individual components, and MACE by quartiles of FGF-23 and levels of hsCRP concentration by randomized treatment assignment. For these analyses, participants were categorized into a “low-risk” (Q1-Q3) or “high-risk” (Q4) group. The interaction term between FGF-23 or hsCRP and randomized treatment assignment were fitted with the Cox proportional hazards model to test for heterogeneity of treatment effect. To compare the absolute differences in the effect of canagliflozin vs placebo on the outcomes of interests according to baseline FGF-23 or baseline hsCRP across each quartile, the absolute risk reduction was calculated by subtracting the event rates of participants assigned to canagliflozin from the event rates of participants assigned to placebo. Participants were then simultaneously stratified by FGF-23 Q1-Q3 vs Q4 and hsCRP Q1-Q3 vs Q4 to create 4 risk groups: a low-low “LL” biomarker-level group (Q1-Q3 FGF-23 + Q1-Q3 hsCRP), a low-high “‘LH” biomarker-level group (Q1-Q3 FGF-23 + Q4 hsCRP), a high-low “HL” group (Q4 FGF-23 + Q1-Q3 hsCRP), and a high-high “HH” group (Q4 FGF-23 + Q4 hsCRP). The unadjusted and adjusted risks were calculated by comparing each of the 4 resulting categories in the overall cohort.

All analyses were performed using R version 3.6.0 (The R Foundation). A 2-sided *P* value of 0.05 was considered significant for all tests.

## Results

### Baseline characteristics

Of 4,330, 3,188 (73.6%) CANVAS trial participants had available baseline FGF-23 and baseline hsCRP values for analysis as presented in Supplemental Table 1. Participants with available biomarker levels were a median age of 63 (IQR: 58-68) years, 1,078 (33.8%) were female, 1,873 (58.8%) had a history of CVD, and 404 (12.7%) had a history of HF. Participant distribution and availability of individual biomarkers at each follow-up by treatment assignment are summarized in Supplemental Figure 1.

Baseline characteristics stratified by FGF-23 quartiles are also summarized in Supplemental Table 1 and baseline characteristics stratified by hsCRP quartiles are summarized in Supplemental Table 2. Unstratified baseline characteristics have been summarized in Supplemental Table 3. The median FGF-23 concentration was 64.8 (IQR: 48.3-97.2) RU/mL. Participants in higher FGF-23 quartiles were older and were more likely to have a history of CVD, MI, microalbuminuria/macroalbuminuria, lower eGFR, and higher NT-proBNP (each *P* < 0.05). The median hsCRP concentration was 2.30 (IQR: 0.90-5.54) mg/L. Participants with higher hsCRP quartiles were more likely to have had a history of stroke, HF, microalbuminuria/macroalbuminuria, and hypertension with higher FGF-23 and NT-proBNP (each *P* < 0.05). Metrics of discrimination (Harrell’s C-statistic) are summarized in Supplemental Table 4.

### Correlation with other biomarkers

FGF-23 levels showed only weak correlations with hsCRP (r = 0.07; *P* < 0.001), UACR (r = 0.07; *P <* 0.005), eGFR (r = −0.13; *P* < 0.001), NT-proBNP (r = 0.15; *P* < 0.001), and hs-cTnT (r = 0.10, *P* < 0.001) as presented in Supplemental Table 5. hsCRP also showed weak correlations with eGFR (r = −0.04; *P* = 0.010) and NT-proBNP (r = 0.06; *P* < 0.001). Longitudinal measurements of FGF-23 and hsCRP at various time points demonstrated that canagliflozin did not change levels of these markers significantly over time (Supplemental Figure 2).

### Association of baseline biomarker levels with cardiovascular outcomes

There were 3,188 participants with available baseline biomarker levels followed for a mean of 307 (±57) weeks, of which 2,112 (66%) participants received canagliflozin while 1,076 (34%) participants received placebo. A total of 308 (9.7%) participants experienced the primary outcome of composite CV death/HHF, 109 (3.4%) participants experienced HHF, 222 (7.0%) participants experienced CV death, and 480 (15%) participants experienced MACE (Supplemental Table 6).

Overall, participants experienced greater risk of the primary and secondary endpoints with increasing quartiles of FGF-23 levels as seen in Table 1. There was a significant upward trend in hazard across quartiles 1 to 4 for the composite of CV death/HHF and HHF from models 1 through 6 (*P* trend ≤0.007 for both). After sequential adjustment for potential confounders in models 1 through 6, participants with the highest quartile of FGF-23 levels were associated with a 65% increased risk for the composite of CV death/HHF (HR: 1.65; 95% CI: 1.15-2.40; *P* = 0.008) and a near doubling of risk for HHF (HR: 1.96; 95% CI: 1.04-3.69; *P* = 0.037). FGF-23 levels in quartile 4 were also associated with CV death (HR: 1.78; 95% CI: 1.17-2.71; *P* = 0.007) after adjustment for potential confounders in models 1 through 5, however this association lost significance once log(hs-cTnT) and log(NT-proBNP) were added to model 6. When considered as a continuous variable, doubling of FGF-23 was significantly associated with a 17% increased risk of composite CV death/HHF (HR: 1.17; 95% CI: 1.04-1.31; *P* = 0.007) and a 34% increased risk of HHF (HR: 1.34; 95% CI: 1.13-1.60; P=<0.001) in the fully adjusted models.

**Table 1:** Associations of Quartiles and Doubling of FGF-23 With Outcomes of Interest in the Overall Cohort

Outcome and Quartile	Model 1			Model 2			Model 3			Model 4			Model 5			Model 6		
	HR (95% CI)	*P* Value	*P* Trend	HR (95% CI)	*P* Value	*P* Trend	HR (95% CI)	*P* Value	*P* Trend	HR (95% CI)	*P* Value	*P* Trend	HR (95% CI)	*P* Value	*P* Trend	HR (95% CI)	*P* Value	*P* Trend
Cardiovascular death or hospitalization for heart failure																		
Quartile 1	(reference)	-	<0.001	(reference)	-	<0.001	(reference)	-	<0.001	(reference)	-	<0.001	(reference)	-	<0.001	(reference)	-	0.007
Quartile 2	0.95 (0.63-1.42)	0.80		0.94 (0.62-1.40)	0.70		0.90 (0.60-1.35)	0.60		0.89 (0.59-1.34)	0.60		0.87 (0.58-1.31)	0.50		0.90 (0.60-1.35)	0.60	
Quartile 3	1.74 (1.22-2.49)	0.002		1.73 (1.21-2.46)	0.003		1.47 (1.02-2.12)	0.038		1.47 (1.02-2.12)	0.038		1.47 (1.02-2.11)	0.040		1.41 (0.97-2.04)	0.070	
Quartile 4	2.98 (2.14-4.15)	<0.001		2.87 (2.05-4.01)	<0.001		2.31 (1.63-3.29)	<0.001		2.17 (1.52-3.10)	<0.001		2.08 (1.46-2.97)	<0.001		1.65 (1.14-2.38)	0.008	
Per doubling	1.40 (1.28-1.53)	<0.001	-	1.41 (1.29-1.54)	<0.001	-	1.34 (1.21-1.48)	<0.001	-	1.32 (1.19-1.46)	<0.001	-	1.30 (1.17-1.45)	<0.001	-	1.17 (1.04-1.31)	0.007	-
Hospitalization for heart failure																		
Quartile 1	(reference)	-	<0.001	(reference)	-	<0.001	(reference)	-	<0.001	(reference)	-	<0.001	(reference)	-	<0.001	(reference)	-	<0.001
Quartile 2	1.12 (0.56-2.23)	0.80		1.13 (0.56-2.26)	0.70		1.08 (0.54-2.16)	0.80		1.08 (0.53-2.16)	0.80		1.06 (0.53-2.14)	0.90		1.07 (0.53-2.16)	0.80	
Quartile 3	1.64 (0.86-3.13)	0.13		1.66 (0.87-3.17)	0.12		1.31 (0.68-2.54)	0.40		1.34 (0.69-2.58)	0.40		1.33 (0.69-2.57)	0.40		1.30 (0.67-2.53)	0.40	
Quartile 4	3.85 (2.17-6.83)	<0.001		3.90 (2.19-6.96)	<0.001		2.78 (1.52-5.11)	<0.001		2.65 (1.43-4.88)	0.002		2.57 (1.39-4.76)	0.003		1.96 (1.04-3.69)	0.037	
Per doubling	1.59 (1.39-1.81)	<0.001	-	1.64 (1.43-1.88)	<0.001	-	1.54 (1.32-1.80)	<0.001	-	1.53 (1.31-1.79)	<0.001	-	1.52 (1.30-1.78)	<0.001	-	1.34 (1.13-1.60)	<0.001	-
Cardiovascular death																		
Quartile 1	(reference)	-	<0.001	(reference)	-	<0.001	(reference)	-	0.004	(reference)	-	0.014	(reference)	-	0.029	(reference)	-	0.80
Quartile 2	1.08 (0.54-2.16)	0.80		0.86 (0.54-1.39)	0.50		0.83 (0.51-1.34)	0.40		0.82 (0.51-1.32)	0.40		0.80 (0.50-1.29)	0.40		0.84 (0.52-1.35)	0.50	
Quartile 3	1.31 (0.68-2.55)	0.40		1.74 (1.16-2.62)	0.008		1.52 (1.00-2.31)	0.051		1.50 (0.99-2.29)	0.057		1.50 (0.98-2.28)	0.060		1.44 (0.94-2.21)	0.10	
Quartile 4	2.78 (1.52-5.11)	<0.001		2.44 (1.65-3.60)	<0.001		2.04 (1.35-3.09)	<0.001		1.89 (1.25-2.88)	0.003		1.78 (1.17-2.71)	0.007		1.40 (0.93-2.16)	0.13	
Per doubling	1.54 (1.32-1.80)	<0.001	-	1.28 (1.14-1.44)	<0.001	-	1.21 (1.06-1.37)	0.004	-	1.18 (1.03-1.35)	0.014	-	1.16 (1.02-1.33)	0.029	-	1.02 (0.88-1.18)	0.80	-
MACE																		
Quartile 1	(reference)	-	0.015	(reference)	-	0.013	(reference)	-	0.20	(reference)	-	0.40	(reference)	-	0.50	(reference)	-	0.50
Quartile 2	0.84 (0.64-1.11)	0.20		0.84 (0.63-1.11)	0.20		0.82 (0.62-1.08)	0.20		0.82 (0.62-1.08)	0.20		0.81 (0.61-1.07)	0.14		0.79 (0.59-1.05)	0.10	
Quartile 3	1.23 (0.95-1.59)	0.11		1.23 (0.95-1.59)	0.11		1.13 (0.87-1.46)	0.40		1.10 (0.85-1.44)	0.50		1.10 (0.84-1.43)	0.50		1.10 (0.84-1.43)	0.50	
Quartile 4	1.47 (1.15-1.88)	0.002		1.45 (1.13-1.86)	0.004		1.28 (0.98-1.67)	0.074		1.22 (0.93-1.59)	0.20		1.19 (0.90-1.55)	0.20		1.01 (0.76-1.34)	>0.90	
Per doubling	1.11 (1.02-1.21)	0.015	-	1.12 (1.02-1.22)	0.013	-	1.06 (0.97-1.17)	0.20	-	1.05 (0.95-1.15)	0.30	-	1.04 (0.94-1.14)	0.50	-	0.97 (0.87-1.07)	0.50	-

Models are adjusted for the following covariates: Model 1: Randomized treatment assignment. Model 2: Covariate of model 1 + age and sex. Model 3: Covariates of model 2 + race, myocardial infarction, stroke, heart failure, type 2 diabetes duration, smoking, hypertension, body mass index, high-density lipoprotein, low-density lipoprotein, estimated glomerular filtration rate. Model 4: Covariates of model 3 + urine albumin-to-creatinine ratio. Model 5: Covariates of model 4 +log(hsCRP). Model 6: Covariates of model 5 + log(hsTnT) and log (NT-proBNP).

FGF-23 = fibroblast growth factor 23; MACE = major adverse cardiovascular events.

With increasing quartiles of hsCRP, participants experienced greater risk of the primary and secondary endpoints as seen in Table 2. There was a significant upward trend in hazard across quartiles 1 to 4 for CV death from models 1 through 6 (*P* trend ≤0.017). After sequential adjustment for potential confounders in models 1 through 6, participants with the highest quartile of hsCRP levels had a 78% increased risk of CV death (HR: 1.78; 95% CI: 1.16-2.73; *P* = 0.008) and a 35% increased risk of MACE (HR: 1.35; 95% CI: 1.02-1.78; *P* = 0.038). hsCRP levels in quartile 4 were also associated with a 68% increased risk of the composite of CV death/HHF (HR: 1.68; 95% CI: 1.18-2.37; *P* = 0.004) from models 1 through 5, however this association lost significance once log(hs-cTnT) and log(NT-proBNP) were added to model 6. When considered as a continuous variable, doubling of hsCRP was associated with a 9% increased risk of CV death (HR: 1.09; 95% CI: 1.02-1.18; *P* = 0.017) in the fully adjusted models.

**Table 2:** Associations of Quartiles and Doubling of hsCRP With Outcomes of Interest in the Overall Cohort

Outcome and Quartile	Model 1			Model 2			Model 3			Model 4			Model 5			Model 6		
	HR (95% CI)	*P* Value	*P* Trend	HR (95% CI)	*P* Value	*P* Trend	HR (95% CI)	*P* Value	*P* Trend	HR (95% CI)	*P* Value	*P* Trend	HR (95% CI)	*P* Value	*P* Trend	HR (95% CI)	*P* Value	*P* Trend
Cardiovascular death or hospitalization for heart failure																		
Quartile 1	(reference)	-	<0.001	(reference)		<0.001	(reference)	-	0.002	(reference)	-	0.003	(reference)	-	0.010	(reference)	-	0.10
Quartile 2	1.52 (1.08-2.15)	0.017		1.54 (1.09-2.17)	0.014		1.41 (0.99-1.99)	0.055		1.33 (0.94-1.89)	0.11		1.33 (0.93-1.88)	0.11		1.22 (0.85-1.74)	0.30	
Quartile 3	1.28 (0.89-1.83)	0.20		1.34 (0.93-1.91)	0.11		1.11 (0.77-1.60)	0.60		1.04 (0.72-1.51)	0.80		1.00 (0.69-1.44)	>0.90		0.96 (0.66-1.40)	0.80	
Quartile 4	2.09 (1.51-2.91)	<0.001		2.39 (1.71-3.33)	<0.001		1.86 (1.32-2.63)	<0.001		1.78 (1.26-2.52)	0.001		1.68 (1.18-2.37)	0.004		1.36 (0.96-1.94)	0.085	
Per doubling	1.13 (1.06-1.19)	<0.001	-	1.15 (1.09-1.22)	<0.001	-	1.10 (1.04-1.17)	0.002	-	1.10 (1.03-1.17)	0.003	-	1.08 (1.02-1.15)	0.010	-	1.05 (0.99-1.12)	0.10	-
Hospitalization for heart failure																		
Quartile 1	(reference)	-	0.021	(reference)	-	0.003	(reference)	-	0.20	(reference)	-	0.20	(reference)	-	0.50	(reference)	-	>0.90
Quartile 2	1.39 (0.80-2.40)	0.20		1.43 (0.82-2.48)	0.20		1.23 (0.70-2.16)	0.50		1.15 (0.66-2.03)	0.60		1.11 (0.60-1.85)	0.70		1.03 (0.58-1.81)	>0.90	
Quartile 3	0.89 (0.48-1.65)	0.70		0.96 (0.52-1.78)	>0.90		0.72 (0.39-1.36)	0.30		0.67 (0.36-1.27)	0.20		0.62 (0.29-1.03)	0.14		0.63 (0.33-1.20)	0.20	
Quartile 4	1.85 (1.10-3.13)	0.021		2.28 (1.34-3.88)	0.002		1.56 (0.89-2.73)	0.12		1.46 (0.83-2.57)	0.20		1.25 (0.58-1.79)	0.40		0.96 (0.53-1.71)	0.90	
Per doubling	1.12 (1.02-1.23)	0.021	-	1.16 (1.05-1.27)	0.003	-	1.07 (0.97-1.19)	0.20	-	1.07 (0.96-1.19)	0.20	-	1.04 (0.94-1.15)	0.50	-	1.00 (0.91-1.11)	>0.90	-
Cardiovascular death																		
Quartile 1	(reference)	-	<0.001	(reference)	-	<0.001	(reference)	-	<0.001	(reference)	-	0.001	(reference)	-	0.002	(reference)	-	0.017
Quartile 2	1.66 (1.09-2.52)	0.019		1.65 (1.09-2.52)	0.019		1.55 (1.01-2.37)	0.044		1.45 (0.95-2.23)	0.088		1.45 (0.94-2.23)	0.090		1.38 (0.89-2.13)	0.20	
Quartile 3	1.55 (1.01-2.38)	0.044		1.60 (1.04-2.45)	0.031		1.44 (0.93-2.22)	0.10		1.37 (0.88-2.12)	0.20		1.34 (0.86-2.08)	0.20		1.28 (0.82-2.00)	0.30	
Quartile 4	2.31 (1.55-3.44)	<0.001		2.59 (1.73-3.88)	<0.001		2.24 (1.47-3.40)	<0.001		2.14 (1.41-3.26)	<0.001		2.08 (1.37-3.17)	<0.001		1.78 (1.16-2.73)	0.008	
Per doubling	1.13 (1.06-1.21)	<0.001	-	1.16 (1.08-1.24)	<0.001	-	1.13 (1.05-1.22)	<0.001	-	1.13 (1.05-1.21)	0.001	-	1.12 (1.04-1.21)	0.002	-	1.09 (1.02-1.18)	0.017	-
MACE																		
Quartile 1	(reference)	-	0.003	(reference)	-	<0.001	(reference)	-	0.008	(reference)	-	0.011	(reference)	-	0.013	(reference)	-	0.055
Quartile 2	1.30 (1.00-1.69)	0.053		1.33 (1.02-1.73)	0.035		1.27 (0.97-1.66)	0.077		1.26 (0.95-1.65)	0.093		1.26 (0.96-1.64)	0.10		1.21 (0.92-1.59)	0.20	
Quartile 3	1.16 (0.89-1.53)	0.30		1.22 (0.93-1.60)	0.20		1.11 (0.84-1.47)	0.50		1.10 (0.83-1.45)	0.50		1.09 (0.82-1.44)	0.50		1.10 (0.82-1.46)	0.50	
Quartile 4	1.53 (1.18-1.98)	0.001		1.73 (1.33-2.25)	<0.001		1.50 (1.14-1.96)	0.004		1.47 (1.12-1.94)	0.005		1.46 (1.11-1.92)	0.006		1.35 (1.02-1.78)	0.038	
Per doubling	1.07 (1.02-1.12)	0.003	-	1.09 (1.04-1.14)	<0.001	-	1.07 (1.02-1.12)	0.008	-	1.06 (1.01-1.11)	0.011	-	1.06 (1.01-1.11)	0.013	-	1.05 (1.00-1.10)	0.055	-

Models are adjusted for the following covariates: Model 1: Randomized treatment assignment. Model 2: Covariate of model 1 + age and sex. Model 3: Covariates of model 2 + race, myocardial infarction, stroke, heart failure, type 2 diabetes duration, smoking, hypertension, body mass index, high-density lipoprotein, low-density lipoprotein, estimated glomerular filtration rate. Model 4: Covariates of model 3 + urine albumin-to-creatinine ratio. Model 5: Covariates of model 4 +log(hsCRP). Model 6: Covariates of model 5 + log(hsTnT) and log (NT-proBNP).

hsCRP = high-sensitivity C-reactive protein; other abbreviation as in Table 1.

### Treatment effect of canagliflozin by baseline biomarker levels

**Figure 1:**
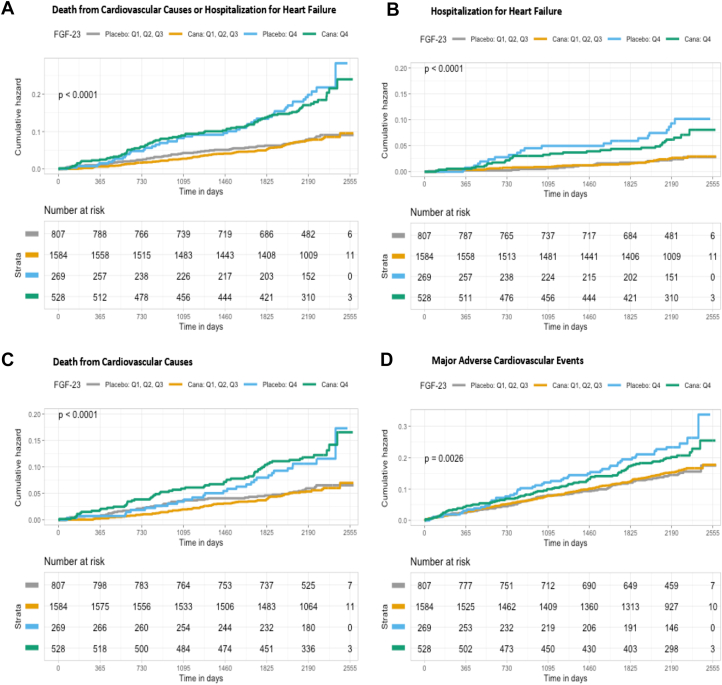
**Kaplan-Meier Event Rates for Cardiovascular Outcomes by High-Risk or Low-Risk Group and Randomized Treatment** High-risk group was defined as FGF-23 levels in Q4. Low-risk group was defined as FGF-23 levels in Q1-Q3. Figures A, B, C, D show that high risk participants, according to FGF-23 levels, were at an increased risk for the composite of CV death/HHF, HHF, CV Death, and MACE, respectively, in both treatment arms compared to the low-risk participants in the unadjusted model. Cana = canagliflozin; CV = cardiovascular; FGF = fibroblast growth factor; HHF = hospitalization for heart failure; MACE = major adverse cardiovascular events.

**Figure 2:**
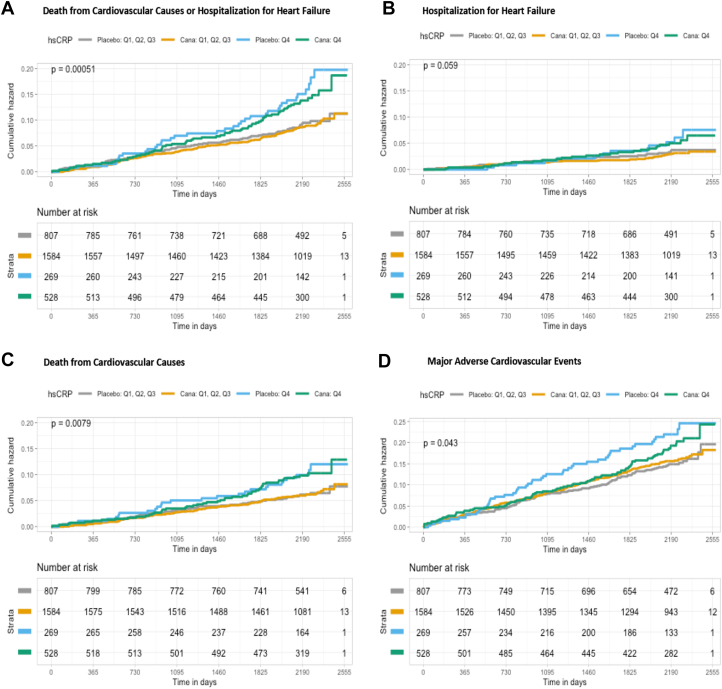
**Kaplan-Meier Event Rates for Cardiovascular Outcomes by High-Risk or Low-Risk Group and Randomized Treatment** High-risk group was defined as hsCRP levels in Q4. Low-risk group was defined as hsCRP levels in Q1-Q3. Figures A, B, C, D show that high risk participants, according to hsCRP levels, were at an increased risk for the composite of CV death/HHF, HHF, CV Death, and MACE, respectively, in both treatment arms compared to the low-risk participants in the unadjusted model. CV = cardiovascular; HHF = hospitalization for heart failure; hsCRP = high-sensitivity C-reactive protein; MACE = major adverse cardiovascular events; other abbreviation as in Figure 1.

Three-year Kaplan-Meier event rates for the composite of CV death/HHF, its individual components, and MACE were highest among participants with FGF-23 levels in Q4 compared with those with FGF-23 levels in Q1-Q3 (Figure 1). This was similar among participants with Q4 hsCRP levels in comparison to Q1-Q3 hsCRP levels (Figure 2).

Participants were classified as high risk if they had Q4 biomarker levels and low risk if they had Q1-Q3 biomarker levels. Regardless of treatment assignment, the high-risk group according to FGF-23 levels had a > 2-fold increased event rate for composite CV death/HHF compared to the low-risk group (Supplemental Table 7). Models testing for interaction revealed that the effect of canagliflozin did not significantly differ between the low- and high-risk group for composite CV death/HHF, individual components, or MACE (*P* for heterogeneity ≥0.30 for all). Similarly, the high-risk group according to hsCRP levels had a > 1.5-fold increased event rate for the composite of CV death/HHF compared to the low-risk group (Supplemental Table 8). Formal interaction testing again revealed a *P* for heterogeneity ≥0.30 for all endpoints suggesting baseline levels of hsCRP did not modify the effect of canagliflozin.

### Multimarker analysis categorized by four risk groups

Participants were simultaneously stratified by FGF-23 and hsCRP levels to create 4 risk groups and adjusted for risk factors in model 6, excluding log(FGF-23) or log(hsCRP). The “HH” group consisting of FGF-23 and hsCRP levels in Q4 had the greatest event rate out of the 4 risk groups, regardless of treatment assignment, for all 4 endpoints with a significant upward trend (*P* < 0.001) going from LL (both FGF-23 and hsCRP levels in Q1-Q3) to LH (FGF-23 levels in Q1-Q3 and hsCRP levels in Q4) to HL (FGF-23 levels in Q4 and hsCRP levels in Q1-Q3) to HH (Table 3).

**Table 3:** Treatment Effect of Canagliflozin on Endpoints by FGF-23 and hsCRP Levels

Group	Event Rate n/N (%)		HR (95% CI)	*P* for Heterogeneity	*P* for Trend
	Placebo	Canagliflozin			
CV death or HHF					
LL	45/630 (7.1%)	80/1,233 (6.5%)	0.89 (0.62-1.28)	0.70	<0.001
LH	16/177 (9.0%)	36/351 (10%)	1.09 (0.60-1.96)		
HL	25/177 (14%)	53/351 (15%)	1.08 (0.67-1.74)		
HH	21/92 (23%)	32/177 (18%)	0.66 (0.38-1.15)		
HHF					
LL	14/630 (2.2%)	25/1,233 (2.0%)	0.89 (0.46-1.71)	0.60	<0.001
LH	4/177 (2.3%)	14/351 (4.0%)	1.67 (0.55-5.07)		
HL	12/177 (6.8%)	20/351 (5.7%)	0.85 (0.41-1.73)		
HH	9/92 (9.8%)	11/177 (6.2%)	0.53 (0.22-1.29)		
CV death					
LL	33/630 (5.2%)	60/1,233 (4.9%)	0.91 (0.60-1.40)	0.40	<0.001
LH	14/177 (7.9%)	26/351 (7.4%)	0.91 (0.47-1.74)		
HL	16/177 (9.0%)	37/351 (11%)	1.20 (0.67-2.16)		
HH	12/92 (13%)	24/177 (14%)	0.98 (0.49-1.96)		
MACE					
LL	80/630 (13%)	171/1,233 (14%)	1.09 (0.84-1.42)	0.20	<0.001
LH	27/177 (15%)	53/351 (15%)	0.95 (0.60-1.52)		
HL	31/177 (18%)	57/351 (16%)	0.93 (0.60-1.44)		
HH	23/92 (25%)	38/177 (21%)	0.71 (0.42-1.19)		

CV death = cardiovascular death; HH = Q4 FGF-23 + Q4 hsCRP; HHF = hospitalization for heart failure; HL = Q4 FGF-23 + Q1-Q3 hsCRP; LH = Q1-Q3 FGF-23 + Q4 hsCRP; LL = Q1-Q3 FGF-23 + Q1-Q3 hsCRP; other abbreviations as in Tables 1 and 2.

**Figure 3:**
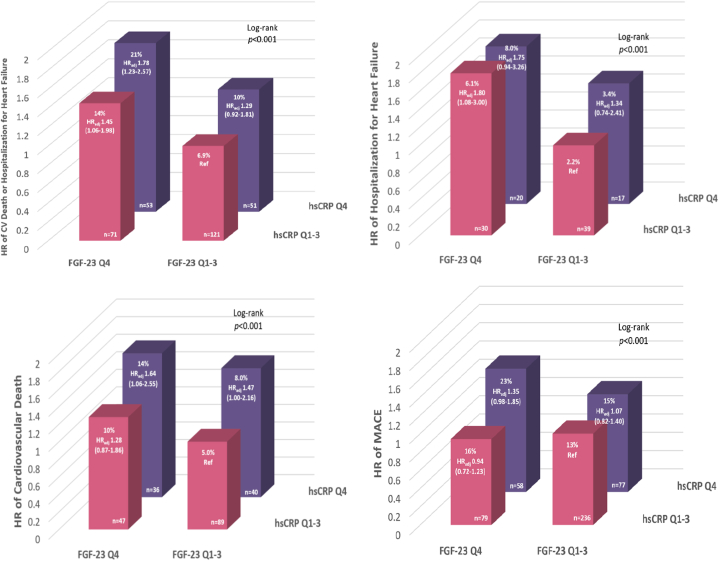
**Adjusted Risk of Cardiovascular Events in the Overall Cohort Stratified by FGF-23 and hsCRP Concentrations** Adjusted covariates: Randomized treatment assignment, age, sex, race, myocardial infarction, stroke, heart failure, type 2 diabetes duration, smoking, hypertension, body mass index, high-density lipoprotein, low-density lipoprotein, estimated glomerular filtration rate, urine albumin-to-creatinine ratio, high-sensitivity cardiac troponin T, and N-terminal pro–B-type natriuretic peptide. CV = cardiovascular; MACE = major adverse cardiovascular events; Q = quartile; other abbreviations as in Figures 1 and 2.

The 3-year Kaplan-Meier event rate for the composite of CV death/HHF was 3-fold higher in participants with both FGF-23 and hsCRP levels in Q4 (n = 53) with a 78% increased risk in CV death/HHF in comparison to the reference group, which are participants with both FGF-23 and hsCRP levels in Q1-Q3 (n = 121; 21% vs 6.9%, respectively; Figure 3). Similar findings were observed for HHF, CV death, and MACE with the “HH” group having the greatest risk out of the 4 risk groups in the fully adjusted model.

## Discussion

In this biomarker substudy of the CANVAS trial among participants with T2DM who were at a high risk of or had established ASCVD, higher circulating FGF-23 was associated with an increased risk of the composite of CV death/HHF and HHF whereas higher circulating hsCRP was associated with an increased risk of CV death and MACE, independent of clinical risk factors, and traditional CV and renal biomarkers. Moreover, participants categorized into the high-risk group had higher event rates and distinctly greater risk than participants categorized into the low-risk group, irrespective of treatment assignment, for the composite of CV death/HHF, individual components, and MACE. There was, however, no significant association between baseline levels of FGF-23 or hsCRP and the effect of canagliflozin on the risk of any of the 4 outcomes of interest. Although FGF-23 and hsCRP were associated with CV risk and canagliflozin-reduced CV events, the drug did not significantly change FGF-23 or hsCRP levels, nor did individuals in the highest baseline quartiles for these biomarkers derive greater benefit. This suggests canagliflozin’s CV benefits may occur through pathways unrelated to inflammation or mineral metabolism. Overall, evidence regarding the effects of SGLT2 inhibitors on inflammation has been inconsistent. In DAPA-HF (Dapagliflozin and Prevention of Adverse Outcomes in Heart Failure) trial, elevated baseline hsCRP predicted higher risk of HF and CV death, yet dapagliflozin did not reduce hsCRP.^7^ In other CANVAS analyses, canagliflozin modestly lowered growth differentiation factor-15, but this did not explain its protective effects on CV, HF, and kidney outcomes^8^; in CREDENCE (Canagliflozin and Renal Events in Diabetes with Established Nephropathy Clinical Evaluation) trial, canagliflozin slowed growth differentiation factor-15 increases over time but also attenuated levels of NT-proBNP, hs-cTnT, and insulin-like growth factor binding protein 7.^9^

Canagliflozin also has been shown to attenuate rises in soluble suppression of tumorigenicity 2 and markers of tubular injury (KIM-1), and fibrosis (TNFR1, TNFR2), which may reflect more direct hemodynamic and renal effects than FGF-23.^9^, ^10^, ^11^ In another trial, canagliflozin lowered NT-proBNP and hs-cTnT I, but not soluble suppression of tumorigenicity 2.^12^ In EMPA-REG OUTCOME (Empagliflozin, Cardiovascular Outcome Event Trial in Type 2 Diabetes Mellitus Patients), empagliflozin reduced hsCRP by 43% over time.^13^ Collectively, these data along with ours underscore variability in SGLT2 inhibitors’ effects on hsCRP and other inflammatory and fibrosis biomarkers.

It had been previously confirmed that elevated levels of FGF-23 and hsCRP, individually, are associated with greater risk of CV events. Our findings confirm and expand on this to a patient population with T2DM and are at high risk for ASCVD. To our knowledge, this analysis is the largest to examine the longitudinal effect of SGLT2i on the trajectories of circulating FGF-23 levels, hsCRP levels.^5^^,^^14^^,^^15^ Additionally, our findings in conjunction with earlier studies emphasize the prognostic value of FGF-23 and hsCRP for adverse CV outcomes.^2^^,^^3^

The pathophysiological mechanisms leading to the production of FGF-23 and hsCRP in humans are distinct. The role of inflammation in the pathophysiology of CVD has been well established and is reflected at least in part by hsCRP levels, an acute phase protein that increases with systemic inflammation.^3^^,^^14^ Numerous prospective studies have shown that hsCRP is an independent predictor of future CV events and this is consistent with what we observed in our results.^3^^,^^14^ We have shown that, independent of traditional risk factors, high levels of hsCRP are associated with MACE and death by CV causes. In the setting of primary prevention, it has been demonstrated that measurement of hsCRP is as impactful as LDL, high-density lipoprotein, total cholesterol, and other traditional risk factors.^16^ It has also been seen that the addition of measuring hsCRP, alongside these traditional risk factors in patients receiving statin therapy after acute coronary syndrome, significantly improved CV risk prediction.^16^ However, our study was not able to demonstrate this aspect in patients with T2DM with or at risk of CVD receiving canagliflozin. This could be due to differences in therapies and patient populations. Prior data have supported the ability of hsCRP in reclassifying individuals considered as intermediate risk, which are those with a Framingham 10-year risk between 5% and 20%, into the appropriate high- or low-risk category.^17^ The JUPITER (Justification for the Use of Statins in Prevention: an Intervention Trial Evaluating Rosuvastatin) trial further exhibited that rosuvastatin reduced MACE among intermediate-risk individuals with low or moderate LDL but with elevated hsCRP (>2 mg/L).^17^ Although we did not observe particularly greater benefit from canagliflozin in our patient population with elevated hsCRP levels, our results support the potential clinical utility of measuring hsCRP for future risk prediction^3^^,^^16^^,^^18^

FGF-23, although not performed in routine clinical practice even though previous studies have demonstrated that risk stratification by FGF-23 levels, can be a useful measurement for improving risk assessment in patients for CV events and mortality.^2^ Although we have contributed to the growing body of evidence demonstrating that elevated FGF-23 is independently associated with CV events, there remains a lack of evidence on the role FGF-23 plays in cardiac damage. Moreover, despite the fact that FGF-23 has been viewed as a biomarker of adverse CV risk, some studies have postulated whether FGF-23 may directly induce cardiac injury. Current data suggest that FGF-23, which can be secreted from cardiac myocytes, can stimulate pro-fibrotic factors and subsequently cardiac fibrosis in a paracrine manner due to the induction of fibrosis-related pathways.^19^ It has also been suggested FGF-23 can promote the progression of left ventricular hypertrophy by inducing pro-hypertrophic genes directly through autocrine pathways.^19^ Additionally, studies in rodents and human cardiac myocytes suggest that the endogenous expression and secretion of FGF-23 by infiltrating macrophages can induce inflammation and fibrosis leading to the progression of CVD.^19^ Therefore, combining measurements of both biomarkers provides a more comprehensive understanding of CV risk pathophysiology, revealing aspects not captured by either biomarker alone, which may account for the elevated risk observed when both are measured simultaneously. It can also be postulated that the increased CV risk seen when measuring the combination of high levels of FGF-23 and hsCRP is simply due to the greater predictive value attained by looking at 2 high-risk biomarkers together rather than one separately.^20^

The results from our study suggest there may be a role to targeting inflammation to reduce CV risk. The CANTOS (Canakinumab Antiinflammatory Thrombosis Outcome Study) demonstrated favorable CV effects in patients with ASCVD through canakinumab, a monoclonal antibody that targets interleukin-1β, a cytokine that is central to the inflammatory response.^21^ Promising data were also seen in the POSIBIL6 Phase 2b trial in patients with end-stage kidney disease showing a reduction in inflammatory biomarkers associated with CV events when treated with an interleukin-6 inhibitor (clazakizumab).^22^ Furthermore, in the COLCOT (Colchicine Cardiovascular Outcomes Trial) colchicine, an anti-inflammatory medication that may have effects on cellular adhesion molecules, inflammatory chemokines, and the inflammasome along with causing the inhibition of tubulin polymerization and microtubule generation, had sizeable reductions in the risk of CV events in patients who had recently had a MI though results have not been consistent in all trials of colchicine in this population.^23^ Whether other therapies that inhibit inflammation, such as ziltivekimab, will show significant cardiorenal benefits remains under investigation.

## Conclusions

In a population with type 2 diabetes who are at high risk or have established CVD, participants with high levels of FGF-23 were associated with an increased risk of the composite of CV death/HHF and the individual endpoint HHF, whereas participants with high levels of hsCRP were associated with an increased risk of CV death and MACE, independent of clinical risk factors and traditional CV or renal biomarkers as summarized in the Central Illustration. Furthermore, the combination of elevated FGF-23 and hsCRP levels identified participants at a distinctly greater risk for adverse CV outcomes. Finally, participants with the highest levels of hsCRP and FGF-23 did not derive a greater benefit from treatment with the canagliflozin.

**Central Illustration:**
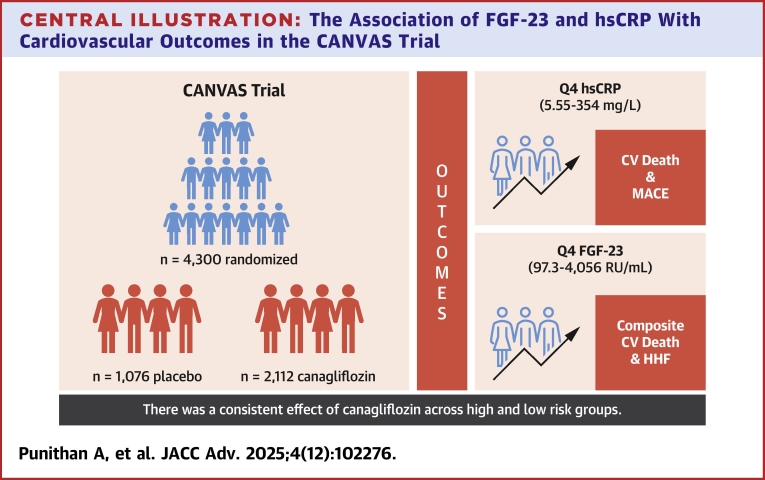
**The Association of FGF-23 and hsCRP With Cardiovascular Outcomes in the CANVAS Trial** FGF-23 levels in Q4 were significantly associated with the composite of CV death/HHF while hsCRP levels in Q4 were significantly associated with CV death and MACE. There was consistent effect of canagliflozin vs placebo across high- and low-risk groups. CANVAS = Canagliflozin Cardiovascular Assessment Study; HHF = hospitalization for heart failure; other abbreviations as in Figure 1, Figure 2, Figure 3.

### Study Limitations

There are several limitations worthy of mention. First, this analysis was post hoc. Associations between biomarkers and outcomes of interest should therefore be regarded as hypothesis generating. Second, the generalizability of the results is limited given that the eligibility criteria of the CANVAS trial was restricted to participants with T2DM with or at high risk of CVD. Third, due to the low incidence of events, these analyses lacked power to detect between-group differences and thus it could not be conclusively determined that canagliflozin had no significant effect on our outcomes of interest based on FGF-23 and hsCRP levels. Fourth, we did not analyze hsCRP levels based on clinically established cutoff ranges, however, our results were consistent with prior findings in the literature.^3^^,^^18^ Fifth, the presence of chronic kidney disease (CKD) at baseline may influence the relationship between canagliflozin and levels of FGF-23 and hsCRP; this may be evaluated in SGLT2 inhibitor trials among patients with CKD. Sixth, this study measured C-terminal FGF-23; its possible associations with intact FGF-23 may differ. Finally, we did not have longitudinal data on all of the clinical markers that we adjusted for and we did not have measurements of mineral metabolism markers, such as phosphate, 1,25-dihydroxyvitamin D_3_, and parathyroid hormone, which causes potential for residual confounding. These are important markers related to FGF-23 levels which could have provided us with more insight on whether adjusting for these markers in our clinical model would have reduced the associations with adverse CV outcomes that we observed.

## Funding support and author disclosures

The CANVAS study was sponsored by 10.13039/100005205Janssen Research & Development. Dr Cherney has received honoraria from Boehringer Ingelheim-Lilly, Merck, AstraZeneca, Sanofi, Mitsubishi-Tanabe, Abbvie, Janssen, AMGEN, Bayer, Prometic, BMS, Maze, Gilead, CSL-Behring, Otsuka, Novartis, Youngene, Lexicon, Inversago, GSK, and Novo Nordisk; and has received operational funding for clinical trials from Boehringer Ingelheim-Lilly, Merck, Janssen, Sanofi, AstraZeneca, CSL-Behring and Novo Nordisk, and Bayer. Dr Billia has received funding for physician-initiated research from Abbott Laboratories. Dr Udell has served on the advisory boards of Boehringer Ingelheim, GlaxoSmithKline, Novavax, Novo Nordisk, Sanofi; has received speaker honoraria from Amgen, AstraZeneca, Boehringer Ingelheim, Eli Lilly and Company; and has received research funding to his institution from Amgen, Bayer, Boehringer Ingelheim, Janssen, and Novartis. Dr Connelly has received funding from 10.13039/100004325AstraZeneca, 10.13039/100001003Boehringer Ingelheim, Eli Lilly, Janssen, Merck, Novartis, Novo Nordisk, Sanofi, Servier, CHRC, GSK, and Abbott. Dr Neal has received funding from Janssen Research Grant to his institution. All other authors have reported that they have no relationships relevant to the contents of this paper to disclose.
