# Supplementary material for: FGF-23, hsCRP, Cardiovascular Events, and the Benefit of Canagliflozin in the CANVAS Trial
Source: JACC Adv. 2025 Oct 27;4(12):102276. doi: 10.1016/j.jacadv.2025.102276 (PMC12596612; doi:10.1016/j.jacadv.2025.102276)
Supplement: Supplemental Tables 1-8 and Figures 1 and 2 [file mmc1.docx]

**SUPPLEMENTAL MATERIAL:**

**Supplemental Table 1. Baseline Characteristics Stratified by FGF-23 Quartiles in the Overall Cohort.**

| **Characteristic** | **Overall**  **n = 3,188** | **FGF-23 Quartile (RU/mL)** | | | | ***P***  **for trend** |
| --- | --- | --- | --- | --- | --- | --- |
|  |  | **Q1**  **(12.1-48.2)**  **(n = 797)** | **Q2**  **(48.3-64.8)**  **(n = 797)** | **Q3**  **(64.9-97.2)**  **(n = 797)** | **Q4**  **(97.3-4056)**  **(n = 797)** |  |
| Demographics |  |  |  |  |  |  |
| Age (years) | 63 (58, 68) | 62 (57, 67) | 63 (57, 68) | 63 (57, 68) | 64 (59, 70) | <0.001 |
| Female sex, *n* (%) | 1,078 (33.8%) | 216 (27.1%) | 259 (32.5%) | 260 (32.6%) | 343 (43.0%) | <0.001 |
| Race, *n* (%) |  |  |  |  |  |  |
| White | 2,610 (81.9%) | 646 (81.1%) | 647 (81.2%) | 673 (84.4%) | 644 (80.8%) | 0.025 |
| Asian | 296 (9.3%) | 76 (9.5%) | 73 (9.2%) | 69 (8.7%) | 78 (9.8%) | 0.081 |
| Black | 75 (2.4%) | 13 (1.6%) | 16 (2.0%) | 18 (2.3%) | 28 (3.5%) | 0.002 |
| Other | 207 (6.5%) | 62 (7.8%) | 61 (7.7%) | 37 (4.6%) | 47 (5.9%) | Ref. |
| Past Medical History, *n* (%) | |  |  |  |  |  |
| CVD | 1,873 (58.8%) | 430 (54.0%) | 454 (57.0%) | 469 (58.8%) | 520 (65.2%) | <0.001 |
| Stroke (TIA) | 545 (17.1%) | 128 (16.1%) | 134 (16.8%) | 139 (17.4%) | 144 (18.1%) | 0.3 |
| MI | 831 (26.1%) | 178 (22.3%) | 208 (26.1%) | 229 (28.7%) | 216 (27.1%) | 0.015 |
| Heart failure | 404 (12.7%) | 98 (12.3%) | 95 (11.9%) | 99 (12.4%) | 112 (14.1%) | 0.3 |
| Diabetes duration (years) | 13 (9, 18) | 12 (8, 17) | 13 (9, 18) | 12 (8, 17) | 14 (9, 20) | <0.001 |
| Micro/macro albuminuria | 839 (26.5%) | 169 (21.2%) | 177 (22.4%) | 223 (28.1%) | 270 (34.2%) | <0.001 |
| Daily cigarette smoker | 570 (17.9%) | 160 (20.1%) | 136 (17.1%) | 140 (17.6%) | 134 (16.8%) | 0.13 |
| Hypertension | 1,359 (42.6%) | 348 (43.7%) | 332 (41.7%) | 346 (43.4%) | 333 (41.8%) | 0.6 |
| Measurements at Enrollment |  |  |  |  |  |  |
| BMI (kg/m^2^) | 32 (28, 36) | 31 (27, 34) | 31 (28, 35) | 32 (29, 37) | 34 (30, 38) | <0.001 |
| HbA1c (%) | 8 (7, 9) | 8 (8, 9) | 8 (7, 9) | 8 (7, 9) | 8 (8, 9) | 0.3 |
| eGFR (mL/min/1.73m^2^) | 76 (65, 89) | 83 (73, 96) | 80 (68, 91) | 74 (64, 86) | 69 (57, 81) | <0.001 |
| UACR (mg/mmol) | 1 (1, 4) | 1 (1, 3) | 1 (1, 3) | 1 (1, 4) | 2 (1, 6) | <0.001 |
| SBP (mmHg) | 136 (126, 146) | 137 (127, 147) | 136 (126, 145) | 137 (126, 146) | 135 (126, 147) | >0.9 |
| DBP (mmHg) | 78 (71, 83) | 79 (73, 85) | 78 (71, 83) | 78 (70, 84) | 75 (69, 82) | <0.001 |
| hsCRP (mg/L) | 2 (1, 6) | 2 (1, 4) | 2 (1, 5) | 2 (1, 5) | 3 (1, 8) | <0.001 |
| hs-cTnT (ng/L) | 12 (8, 17) | 11 (8, 15) | 12 (8, 16) | 12 (8, 18) | 14 (9, 21) | <0.001 |
| NT-proBNP (pg/mL) | 93 (42, 207) | 76 (37, 160) | 78 (38, 180) | 93 (39, 212) | 126 (58, 304) | <0.001 |
| Medical Treatments, *n* (%) |  |  |  |  |  |  |
| Any diuretic | 1,472 (46.2%) | 285 (35.8%) | 329 (41.3%) | 397 (49.8%) | 461 (57.8%) | <0.001 |
| RAAS inhibitor | 2,634 (82.6%) | 639 (80.2%) | 646 (81.1%) | 682 (85.6%) | 667 (83.7%) | 0.012 |
| Statin | 2,358 (74.0%) | 525 (65.9%) | 603 (75.7%) | 607 (76.2%) | 623 (78.2%) | <0.001 |
| Metformin | 2,313 (72.6%) | 616 (77.3%) | 595 (74.7%) | 581 (72.9%) | 521 (65.4%) | <0.001 |
| Insulin | 1,660 (52.1%) | 366 (45.9%) | 393 (49.3%) | 392 (49.2%) | 509 (63.9%) | <0.001 |
| Beta blocker | 1,588 (49.8%) | 340 (42.7%) | 372 (46.7%) | 423 (53.1%) | 453 (56.8%) | <0.001 |
| Sulphonylurea | 1,369 (42.9%) | 360 (45.2%) | 371 (46.5%) | 358 (44.9%) | 280 (35.1%) | <0.001 |
| GLP-1 | 79 (2.5%) | 16 (2.0%) | 18 (2.3%) | 18 (2.3%) | 27 (3.4%) | 0.094 |

Continuous variables are presented as median (interquartile range). Categorical variables are presented as numbers and percentages. CVD, cardiovascular disease; TIA, transient ischemic attack; MI, myocardial infarction; BMI, body mass index; HbA1c, hemoglobin A1C; eGFR, estimate glomerular filtration rate; UACR, urine albumin-to-creatinine ratio; SBP, systolic blood pressure; DBP, diastolic blood pressure; hsCRP, high sensitivity C-reactive protein; hs-cTnT, high sensitivity cardiac troponin T; NT-proBNP, N-terminal pro–B-type natriuretic peptide; RAAS, renin angiotensin aldosterone system; GLP-1, glucagon-like peptide 1.

**Supplemental Table 2. Baseline Characteristics Stratified by hsCRP Quartiles in the Overall Cohort.**

| **Characteristic** | **Overal1**  **n = 3,188** | **hsCRP Quartile (mg/L)** | | | | ***P***  **for trend** |
| --- | --- | --- | --- | --- | --- | --- |
|  |  | **Q1**  **(0.00-0.89)**  **(n = 797)** | **Q2**  **(0.90-2.30)**  **(n = 797)** | **Q3**  **(2.31-5.54)**  **(n = 797)** | **Q4**  **(5.55-354)**  **(n = 797)** |  |
| Demographics |  |  |  |  |  |  |
| Age (years) | 63 (58, 68) | 63 (58, 68) | 63 (58, 69) | 63 (58, 69) | 62 (56, 67) | <0.001 |
| Female sex, *n* (%) | 1,078 (33.8%) | 180 (22.6%) | 245 (30.7%) | 275 (34.5%) | 378 (47.4%) | <0.001 |
| Race, *n* (%) |  |  |  |  |  |  |
| White | 2,610 (81.9%) | 633 (79.4%) | 660 (82.8%) | 653 (81.9%) | 664 (83.3%) | 0.075 |
| Asian | 296 (9.3%) | 80 (10.0%) | 78 (9.8%) | 79 (9.9%) | 59 (7.4%) | 0.843 |
| Black | 75 (2.4%) | 17 (2.1%) | 12 (1.5%) | 21 (2.6%) | 25 (3.1%) | 0.017 |
| Other | 207 (6.5%) | 67 (8.4%) | 47 (5.9%) | 44 (5.5%) | 49 (6.1%) | Ref. |
| Past Medical History, *n* (%) |  |  |  |  |  |  |
| CVD | 1,873 (58.8%) | 503 (63.1%) | 459 (57.6%) | 456 (57.2%) | 455 (57.1%) | 0.018 |
| Stroke (TIA) | 545 (17.1%) | 130 (16.3%) | 119 (14.9%) | 133 (16.7%) | 163 (20.5%) | 0.018 |
| MI | 831 (26.1%) | 233 (29.2%) | 191 (24.0%) | 212 (26.6%) | 195 (24.5%) | 0.093 |
| Heart failure | 404 (12.7%) | 69 (8.7%) | 106 (13.3%) | 105 (13.2%) | 124 (15.6%) | <0.001 |
| Diabetes duration (years) | 13 (9, 18) | 13 (10, 19) | 13 (9, 18) | 12 (8, 17) | 12 (8, 17) | <0.001 |
| Micro/macro albuminuria | 839 (26.5%) | 169 (21.4%) | 216 (27.1%) | 204 (25.8%) | 250 (31.6%) | <0.001 |
| Daily cigarette smoker | 570 (17.9%) | 135 (16.9%) | 139 (17.4%) | 145 (18.2%) | 151 (18.9%) | 0.3 |
| Hypertension | 1,359 (42.6%) | 307 (38.5%) | 326 (40.9%) | 375 (47.1%) | 351 (44.0%) | 0.004 |
| Measurements at Enrollment |  |  |  |  |  |  |
| BMI (kg/m^2^) | 32 (28, 36) | 30 (27, 33) | 31 (28, 35) | 33 (29, 37) | 34 (30, 39) | <0.001 |
| HbA1c (%) | 8 (7, 9) | 8 (7, 9) | 8 (8, 9) | 8 (8, 9) | 8 (8, 9) | <0.001 |
| eGFR (mL/min/1.73m^2^) | 76 (65, 89) | 78 (67, 90) | 76 (65, 88) | 76 (63, 90) | 76 (64, 88) | 0.011 |
| UACR (mg/mmol) | 1 (1, 4) | 1 (1, 3) | 1 (1, 4) | 1 (1, 4) | 2 (1, 5) | 0.008 |
| SBP (mmHg) | 136 (126, 146) | 134 (124, 145) | 136 (127, 145) | 138 (128, 148) | 137 (126, 147) | 0.001 |
| DBP (mmHg) | 78 (71, 83) | 76 (70, 82) | 78 (71, 83) | 79 (72, 84) | 79 (70, 85) | <0.001 |
| FGF-23 (RU/mL) | 65 (48, 97) | 59 (45, 83) | 61 (48, 89) | 68 (50, 102) | 73 (53, 115) | <0.001 |
| hs-cTnT (ng/L) | 12 (8, 17) | 12 (8, 16) | 12 (8, 18) | 12 (8, 18) | 11 (7, 18) | 0.12 |
| NT-proBNP (pg/mL) | 93 (42, 207) | 85 (41, 182) | 90 (41, 193) | 92 (41, 202) | 106 (46, 251) | <0.001 |
| Medical Treatments, *n* (%) |  |  |  |  |  |  |
| Any diuretic | 1,472 (46.2%) | 314 (39.4%) | 347 (43.5%) | 409 (51.3%) | 402 (50.4%) | <0.001 |
| RAAS inhibitor | 2,634 (82.6%) | 665 (83.4%) | 658 (82.6%) | 649 (81.4%) | 662 (83.1%) | 0.7 |
| Statin | 2,358 (74.0%) | 646 (81.1%) | 594 (74.5%) | 579 (72.6%) | 539 (67.6%) | <0.001 |
| Metformin | 2,313 (72.6%) | 602 (75.5%) | 610 (76.5%) | 579 (72.6%) | 522 (65.5%) | <0.001 |
| Insulin | 1,660 (52.1%) | 376 (47.2%) | 411 (51.6%) | 421 (52.8%) | 452 (56.7%) | <0.001 |
| Beta blocker | 1,588 (49.8%) | 408 (51.2%) | 370 (46.4%) | 412 (51.7%) | 398 (49.9%) | 0.8 |
| Sulphonylurea | 1,369 (42.9%) | 362 (45.4%) | 347 (43.5%) | 338 (42.4%) | 322 (40.4%) | 0.039 |
| GLP-1 | 79 (2.5%) | 23 (2.9%) | 18 (2.3%) | 18 (2.3%) | 20 (2.5%) | 0.6 |

Continuous variables are presented as median (interquartile range). Categorical variables are presented as numbers and percentages. CVD, cardiovascular disease; TIA, transient ischemic attack; MI, myocardial infarction; BMI, body mass index; HbA1c, hemoglobin A1C; eGFR, estimate glomerular filtration rate; UACR, urine albumin-to-creatinine ratio; SBP, systolic blood pressure; DBP, diastolic blood pressure; hsCRP, high sensitivity C-reactive protein; hs-cTnT, high sensitivity cardiac troponin T; NT-proBNP, N-terminal pro–B-type natriuretic peptide; RAAS, renin angiotensin aldosterone system; GLP-1, glucagon-like peptide 1.

**Supplemental Figure**
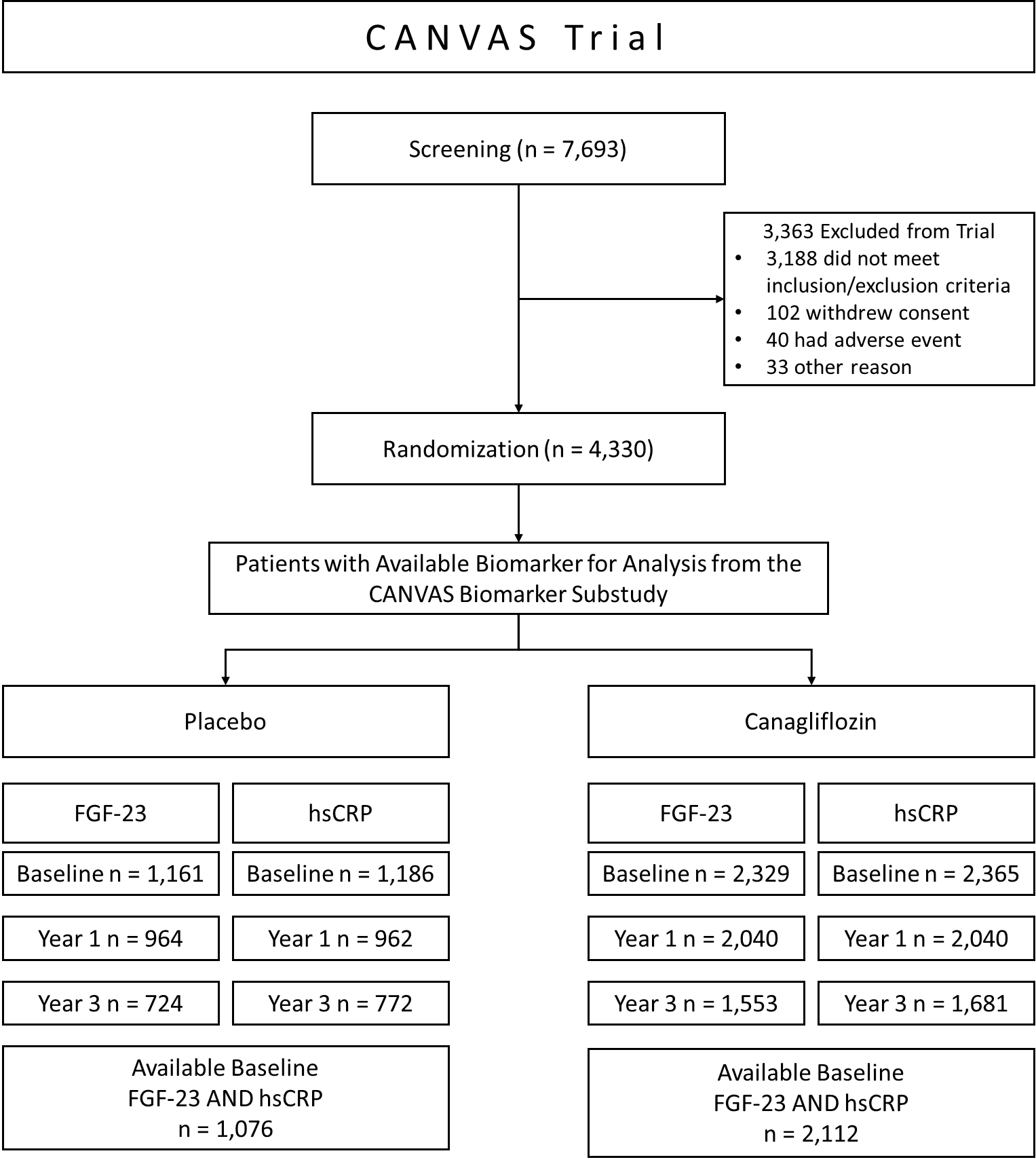
**1. Flowchart of Patient distribution and Availability of Individual Biomarker at Each Follow-Up by Treatment Assignment.**

**Supplemental Table 3. Baseline Characteristics of CANVAS Trial Participants.**

| **Characteristic** | **Overall**  **N = 4,330** | **Biomarker Cohort**  **N = 3,188** |
| --- | --- | --- |
| Demographics |  |  |
| Age (years) | 62 (57, 68) | 63 (58, 68) |
| Female sex, *n* (%) | 1,469 (33.9%) | 1,078 (33.8%) |
| Race, *n* (%) |  |  |
| White | 3,179 (73.4%) | 2,610 (81.9%) |
| Asian | 795 (18.4%) | 296 (9.3%) |
| Black | 105 (2.4%) | 75 (2.4%) |
| Other | 251 (5.8%) | 207 (6.5%) |
| Past Medical History, *n* (%) |  |  |
| CVD | 2,549 (58.9%) | 1,873 (58.8%) |
| Stroke (TIA) | 694 (16.0%) | 545 (17.1%) |
| MI | 1,119 (25.8%) | 831 (26.1%) |
| Heart failure | 515 (11.9%) | 404 (12.7%) |
| Diabetes duration (years) | 12 (8, 18) | 13 (9, 18) |
| Micro/macro albuminuria | 1,218 (28.3%) | 839 (26.5%) |
| Daily cigarette smoker | 776 (17.9%) | 570 (17.9%) |
| Hypertension | 1,846 (42.6%) | 1,359 (42.6%) |
| Measurements at Enrollment |  |  |
| BMI (kg/m^2^) | 31 (28, 36) | 32 (28, 36) |
| HbA1c (%) | 8 (8, 9) | 8 (7, 9) |
| eGFR(mL/min/1.73m^2^) | 76 (65, 89) | 76 (65, 89) |
| UACR (mg/mmol) | 1 (1, 4) | 1 (1, 4) |
| SBP (mmHg) | 136 (126, 146) | 136 (126, 146) |
| DBP (mmHg) | 79 (71, 84) | 78 (71, 83) |
| Medical Treatments, *n* (%) |  |  |
| Any diuretic | 1,901 (43.9%) | 1,472 (46.2%) |
| RAAS inhibitor | 3,490 (80.6%) | 2,634 (82.6%) |
| Statin | 3,131 (72.3%) | 2,358 (74.0%) |
| Metformin | 3,171 (73.2%) | 2,313 (72.6%) |
| Insulin | 2,174 (50.2%) | 1,660 (52.1%) |
| Beta blocker | 2,179 (50.3%) | 1,588 (49.8%) |
| Sulphonylurea | 2,033 (47.0%) | 1,369 (42.9%) |
| GLP-1 | 96 (2.2%) | 79 (2.5%) |
| FGF-23 (RU/mL) | 65 (49, 98) | 65 (48, 97) |
| hsCRP (ng/mL) | 2 (1, 6) | 2 (1, 6) |
| hs-cTnT (ng/L) | 12 (8, 18) | 12 (8, 17) |
| NT-proBNP (pg/mL) | 91 (41, 204) | 93 (42, 207) |

Continuous variables are presented as median (IQR). CVD, cardiovascular disease; TIA, transient ischemic attack; MI, myocardial infarction; BMI, body mass index; HbA1c, hemoglobin A1C; eGFR, estimate glomerular filtration rate; UACR, urine albumin-to-creatinine ratio; SBP, systolic blood pressure; DBP, diastolic blood pressure; RAAS, renin angiotensin aldosterone system; GLP-1, glucagon-like peptide 1.

**Supplemental Figure 2. Trajectory of Biomarker Levels During Treatment with Canagliflozin or Placebo.**


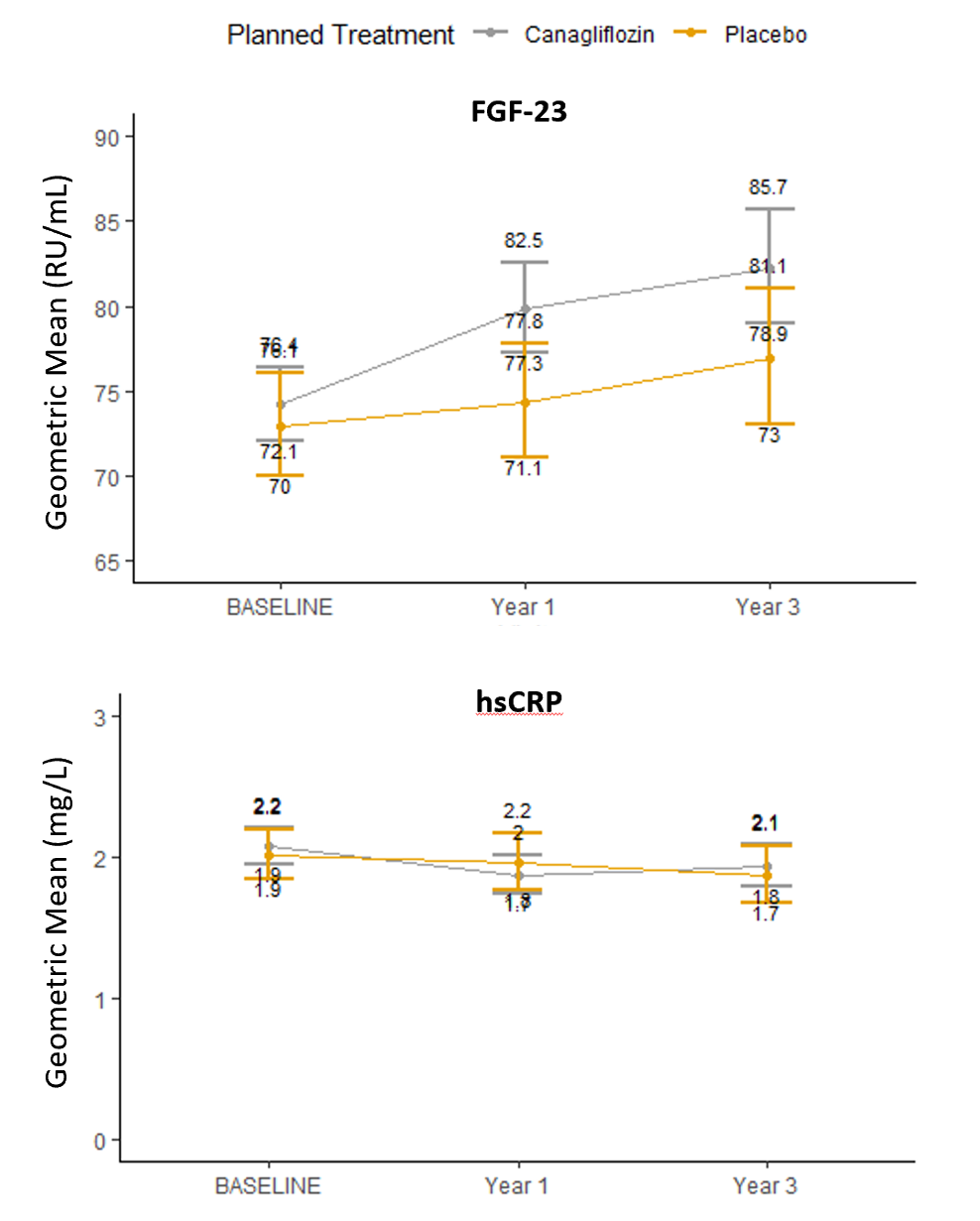

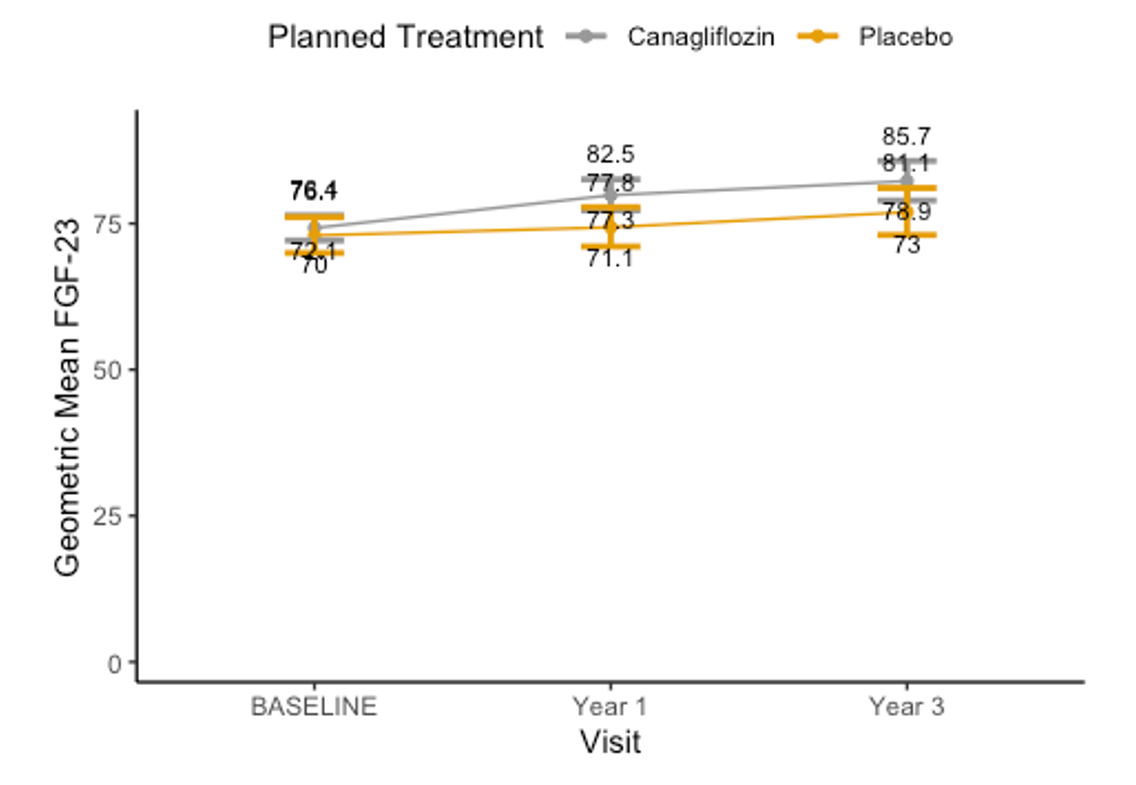


**FGF-23**

The data are presented as geometric mean levels of each biomarker. A linear mixed-model with log(biomarker) as the outcome variable and visit as the predictor was used.

**Supplemental Table 4. C-statistics for All Endpoints.**

| **Model** | **C-Statistics** | **Lower 95% CI** | **Upper 95% CI** | **Likelihood Ratio Test P-values** |
| --- | --- | --- | --- | --- |
| **CV Death or HHF** | | | | |
| Clinical Model | 0.788 | 0.761 | 0.815 | - |
| Clinical Model vs Clinical Model + log2(FGF-23) | 0.790 | 0.763 | 0.817 | 0.005 |
| Clinical Model vs Clinical Model + log2(hsCRP) | 0.789 | 0.761 | 0.816 | 0.058 |
| Clinical Model vs Clinical Model + log2(FGF-23) and log2(CRP) | 0.790 | 0.763 | 0.817 | 0.005 |
| **HHF** | | | | |
| Clinical Model | 0.835 | 0.794 | 0.875 | - |
| Clinical Model vs Clinical Model + log2(FGF-23) | 0.834 | 0.795 | 0.874 | 0.001 |
| Clinical Model vs Clinical Model + log2(hsCRP) | 0.834 | 0.793 | 0.874 | 0.679 |
| Clinical Model vs Clinical Model + log2(FGF-23) and log2(CRP) | 0.834 | 0.795 | 0.874 | 0.005 |
| **CV Death** | | | | |
| Clinical Model | 0.779 | 0.747 | 0.811 | - |
| Clinical Model vs Clinical Model + log2(FGF-23) | 0.780 | 0.747 | 0.812 | 0.639 |
| Clinical Model vs Clinical Model + log2(hsCRP) | 0.783 | 0.751 | 0.815 | 0.015 |
| Clinical Model vs Clinical Model + log2(FGF-23) and log2(CRP) | 0.783 | 0.751 | 0.815 | 0.049 |
| **MACE** | | | | |
| Clinical Model | 0.692 | 0.668 | 0.716 | - |
| Clinical Model vs Clinical Model + log2(FGF-23) | 0.692 | 0.668 | 0.716 | 0.596 |
| Clinical Model vs Clinical Model + log2(hsCRP) | 0.692 | 0.668 | 0.717 | 0.058 |
| Clinical Model vs Clinical Model + log2(FGF-23) and log2(CRP) | 0.692 | 0.668 | 0.717 | 0.135 |
| **KO** | | | | |
| Clinical Model | 0.690 | 0.666 | 0.713 | - |
| Clinical Model vs Clinical Model + log2(FGF-23) | 0.690 | 0.666 | 0.713 | 0.406 |
| Clinical Model vs Clinical Model + log2(hsCRP) | 0.690 | 0.667 | 0.714 | 0.014 |
| Clinical Model vs Clinical Model + log2(FGF-23) and log2(CRP) | 0.690 | 0.667 | 0.714 | 0.037 |

CV death, cardiovascular death; HHF, hospitalization for heart failure; MACE, major adverse cardiovascular events; KO, composite kidney outcomes. Clinical model includes covariates of model 6, excluding log(FGF-23) and log(hsCRP).

**Supplemental Table 5. Spearman’s Correlation Coefficients**

| **Variable** | **FGF-23** | **hsCRP** | **UACR** | **eGFR** | **NT-proBNP** | **hs-cTnT** |
| --- | --- | --- | --- | --- | --- | --- |
| **FGF-23** | - | 0.07 | 0.07 | -0.13 | 0.15 | 0.10 |
| **hsCRP** |  | **-** | 0.03* | -0.04 | 0.06 | 0.03* |
| **UACR** |  |  | - | -0.12 | 0.17 | 0.21 |
| **eGFR** |  |  |  | - | -0.21 | -0.22 |
| **NT-proBNP** |  |  |  |  | - | 0.34 |
| **hs-cTnT** |  |  |  |  |  | - |

***P<*0.05 for all with exception to coefficients marked with ***

FGF-23, fibroblast growth factor 23; hsCRP, high sensitivity C-reactive protein; eGFR, estimated glomerular filtration rate; UACR, urine albumin-to-creatinine ratio; NT-proBNP, N-terminal pro–B-type natriuretic peptide; hs-cTnT, high sensitivity cardiac troponin.

**Supplemental Table 6.** **Overall Event Rates for Outcomes of Interest in Patients with Available Baseline Biomarkers.**

| **Outcome** | **Event Rate**  **n/N (%)** |
| --- | --- |
| CV Death or Hospitalization for Heart Failure | 308/3188 (9.7%) |
| Hospitalization for Heart Failure | 109/3188 (3.4%) |
| CV Death | 222/3188 (7.0%) |
| MACE | 480/3188 (15%) |

CV death, cardiovascular death; MACE, major adverse cardiovascular events.

**Supplemental Table 7. Treatment Effect of Canagliflozin on Endpoints by Low Risk versus High Risk Group of FGF-23 Levels.**

| **Group** | **Event Rate n/N (%)** | | **Hazard ratio**  **(95% CI)** | ***P* for Heterogeneity** |
| --- | --- | --- | --- | --- |
|  | **Placebo** | **Canagliflozin** |  |  |
| **CV Death or HHF** | | | | |
| Low risk | 61/819 (7.4%) | 115/1,572 (7.3%) | 0.96 (0.70-1.31) | 0.60 |
| High risk | 46/257 (18%) | 86/540 (16%) | 0.84 (0.59-1.20) |  |
| **HHF** | | | | |
| Low risk | 18/819 (2.2%) | 38/1,572 (2.4%) | 1.07 (0.61-1.87) | 0.30 |
| High risk | 21/257 (8.2%) | 32/540 (5.9%) | 0.69 (0.40-1.19) |  |
| **CV Death** | | | | |
| Low risk | 47/819 (5.7%) | 38/1,572 (2.4%) | 0.93 (0.65-1.32) | 0.70 |
| High risk | 28/257 (11%) | 62/540 (11%) | 1.05 (0.67-1.64) |  |
| **MACE** | | | | |
| Low risk | 109/819 (13%) | 221/1,572 (14%) | 1.05 (0.83-1.32) | 0.30 |
| High risk | 52/257 (20%) | 98/540 (18%) | 0.84 (0.60-1.18) |  |

The treatment effect Cox models were adjusted for treatment, risk group, and an interaction term for treatment*risk group. Low risk: Quartiles 1-3 FGF-23 levels; High risk: Quartile 4 FGF-23 levels. CV death, cardiovascular death; HHF, hospitalization for heart failure; MACE, major adverse cardiovascular events.

**Supplemental Table 8. Treatment Effect of Canagliflozin on Endpoints by Low Risk versus High Risk Group of hsCRP Levels.**

| **Group** | **Event Rate n/N (%)** | | **Hazard ratio**  **(95% CI)** | ***P* for Heterogeneity** |
| --- | --- | --- | --- | --- |
|  | **Placebo** | **Canagliflozin** |  |  |
| **CV Death or HHF** | | | | |
| Low risk | 70/817 (8.6%) | 132/1,574 (8.4%) | 0.96 (0.72-1.29) | 0.60 |
| High risk | 37/259 (14%) | 69/538 (13%) | 0.82 (0.55-1.23) |  |
| **HHF** | | | | |
| Low risk | 26/817 (3.2%) | 45/1,574 (2/9%) | 0.88 (0.54-1.43) | >0.90 |
| High risk | 13/259 (5.0%) | 25/538 (4.6%) | 0.85 (0.43-1.65) |  |
| **CV Death** | | | | |
| Low risk | 49/817 (6.0%) | 132/1,574 (8.4%) | 1.01 (0.72-1.29) | 0.70 |
| High risk | 26/259 (10%) | 51/538 (9.5%) | 0.82 (0.55-1.23) |  |
| **MACE** | | | | |
| Low risk | 112/817 (14%) | 225/1,574 (14%) | 1.04 (0.83-1.31) | 0.30 |
| High risk | 49/259 (19%) | 94/538 (17%) | 0.85 (0.60-1.20) |  |

The treatment effect Cox models were adjusted for treatment, risk group, and an interaction term for treatment*risk group. Low risk: Quartiles 1-3 hsCRP levels; High risk: Quartile 4 hsCRP levels. CV death, cardiovascular death; HHF, hospitalization for heart failure; MACE, major adverse cardiovascular events.
